# Yield and quality of brown rice noodles processed from early-season rice grains

**DOI:** 10.1038/s41598-021-98352-7

**Published:** 2021-09-21

**Authors:** Min Huang, Zhengwu Xiao, Jiana Chen, Fangbo Cao

**Affiliations:** Crop and Environment Research Center for Human Health, Key Laboratory of Ministry of Education for Crop Physiology and Molecular Biology, Hunan Agricultural University, Changsha, 410128 China

**Keywords:** Plant sciences, Chemistry, Physics

## Abstract

Producing rice noodles using early-season rice grains is a way to bypass difficulties in marketing early-season rice that does not meet consumer preference for soft-textured rice. In recent years, brown rice foods including noodles have attracted great attention due to their health and nutritional benefits. This study was conducted to evaluate the yield and quality of brown rice noodles processed from two early-season rice cultivars. Results showed that the yield of brown rice noodles was 12–19% higher than that of white rice noodles. Although the cooked break rate and cooking loss rate were 5–10% higher in brown rice noodles compared to white rice noodles, both were within an acceptable range for brown rice noodles. Cooked brown rice noodles had 21–27% lower hardness and chewiness than cooked white rice noodles, though differences in the elasticity parameters springiness, cohesiveness, and resilience were not significant or were inconsistent between cooked brown and white rice noodles. These results suggest that it is feasible to process early-season rice to produce brown rice noodles of desirable yield and quality.

## Introduction

Rice is the most important food crop in China, as more than 65% of the population relies on it as a staple food^1^. The development of double-season rice cropping systems (*i.e.*, successively growing early- and late-season rice within a year) in southern China is critical in ensuring food security in China^2^, where the arable land per capita is far below the world average^3^. However, the planting area of double-season rice, particularly early-season rice, has decreased sharply in recent decades^4^. The decrease in planting area of early-season rice is related to improved living standards in China, which has led to demand for and consumption of high-quality rice, especially rice with flavor desired by consumers^5^. Namely, early-season rice generally has a high amylose content, which does not meet consumer preference for rice with low amylose content and soft texture^6^.

Due to concerns about the negative impact of the decreasing planting area of early-season rice on national food security, the Chinese government is trying to reverse the trend of early-season rice planting area decreasing by promoting the sale and consumption of early-season rice. Early-season rice can not only be directly consumed as cooked rice but can also be used to make various food products^7^. Early-season rice with high amylose content is suitable for manufacturing rice noodles^8^, which are a popular and traditional staple food item in southern China^9^. Some southern provinces of China such as Hunan have begun to develop special early-season rice for producing rice noodles in order to effectively market early-season rice.

Rice noodles have been produced using only white rice (milled rice) in China, because convention suggests that the taste of brown rice noodles is not accepted by consumers^10,11^. But in fact, most consumers do not have experience with eating brown rice noodles and have no preconceived notions about them. In recent years, whole grain (including brown rice) foods have attracted great attention due to their health benefits, such as reducing the risk of non-communicable diseases like cardiovascular disease, cancer, and diabetes^12,13^, which are of increasing concern both nationally and globally due to their high mortality rates^14^. In addition, since brown rice undergoes a low degree of milling, milling loss can be reduced, thereby preserving the nutritional composition of rice, such as fat, protein, phosphorus, calcium, and B vitamins^15^.

In this study, we determined the milling recovery rate, starch content, and paste characteristics of brown and white rice as well as the yield, texture, and cooking properties of brown and white rice noodles processed from two early-season rice cultivars. Our objectives were to evaluate the yield and quality of brown rice noodles and provide a basis for further development and utilization of early-season rice.

## Results

**Figure 1:**
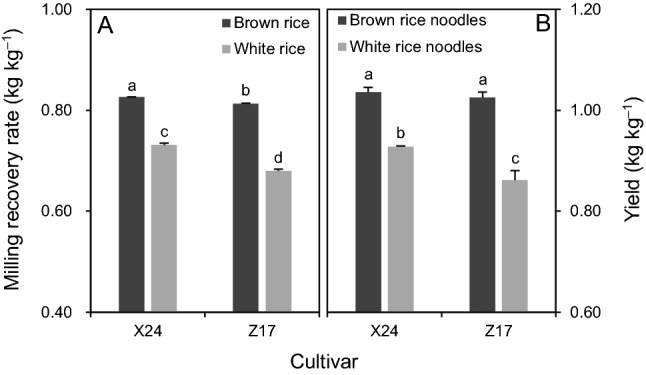
Milling recovery rate of brown and white rice (**A**) and yield of brown and white rice noodles (**B**) processed from two rice cultivars, Xiangzaoxian 24 (X24) and Zhongjiazao 17 (Z17). Data are mean ± SE (*n* = 3). Bars sharing the same lowercase letters are not significantly different at the 0.05 probability level.

There were significant differences between milling recovery rates of brown and white rice and between yields of brown and white rice noodles for both X24 and Z17 (Fig. 1A,B). The milling recovery rates of brown rice were respectively 13% and 20% higher than those of white rice for X24 and Z17 (Fig. 1A). The yields of brown rice noodles were higher than those of white rice noodles by 12% in X24 and 19% in Z17 (Fig. 1B).

The differences in total starch and amylose contents were significant between brown and white rice flours for both X24 and Z17 (Table 1). Total starch contents were 10% and 8% lower in brown rice flour than in white rice flour for X24 and Z17, respectively. Brown rice flour had lower amylose content than white rice flour by 10% for X24 and by 12% for Z17.

**Table 1:** Total starch and amylose content in brown and white rice flours processed from two rice cultivars, Xiangzaoxian 24 (X24) and Zhongjiazao 17 (Z17).

Cultivar	Noodle	Total starch content (g g^−1^)	Amylose content (g g^−1^)
X24	Brown	0.638 ± 0.002d	0.219 ± 0.004c
	White	0.708 ± 0.005b	0.243 ± 0.002b
Z17	Brown	0.677 ± 0.001c	0.243 ± 0.002b
	White	0.733 ± 0.005a	0.275 ± 0.005a

Data are mean ± SE (*n* = 3).

Within a column, data sharing the same lowercase letters are not significantly different at the 0.05 probability level.

There were significant differences in all paste properties, except for consistency viscosity and time to peak viscosity, between brown and white rice flours for both X24 and Z17 (Table 2). Peak, trough, breakdown, and final viscosities were lower in brown rice flour than in white rice flour by 18–37% for X24 and by 24–36% for Z17, while setback viscosity was higher in brown rice flour than in white rice flour by 21% for both X24 and Z17. Paste temperatures were respectively 6.3 and 1.0 °C higher for brown rice flour than for white rice flour for X24 and Z17.

**Table 2:** Paste properties of brown and white rice flours processed from two rice cultivars, Xiangzaoxian 24 (X24) and Zhongjiazao 17 (Z17).

Cultivar	Flour	Viscosity (cP)						Time to peak viscosity (min)	Paste temperature (°C)
		Peak	Trough	Breakdown	Final	Setback	Consistency		
X24	Brown	2002 ± 43b	1640 ± 42c	362 ± 3c	3445 ± 44c	1443 ± 18a	1805 ± 21a	6.31 ± 0.02a	87.1 ± 0.0a
	White	2991 ± 6a	2416 ± 86a	575 ± 80b	4188 ± 41a	1197 ± 35b	1771 ± 62a	6.22 ± 0.08a	80.8 ± 0.0b
Z17	Brown	1866 ± 36b	1343 ± 30d	523 ± 17b	2796 ± 67d	930 ± 32c	1452 ± 46b	5.87 ± 0.04b	81.0 ± 0.3b
	White	2891 ± 66a	2098 ± 62b	793 ± 10a	3658 ± 61b	766 ± 13d	1560 ± 21b	5.91 ± 0.02b	80.0 ± 0.0c

Data are mean ± SE (*n* = 3).

Within a column, data sharing the same lowercase letters are not significantly different at the 0.05 probability level.

There were significant differences in cooking properties between brown and white rice noodles for both X24 and Z17 (Table 3). Cooked break rate was 9% and 10% higher in brown rice noodles than in white rice noodles for X24 and Z17, respectively. Cooking loss rate was higher in brown rice noodles than in white rice noodles by 5% for X24 and by 7% for Z17.

**Table 3:** Cooking properties of brown and white rice noodles processed from two rice cultivars, Xiangzaoxian 24 (X24) and Zhongjiazao 17 (Z17).

Cultivar	Noodle	Cooked break rate (%)	Cooking loss rate (%)
X24	Brown	11.1 ± 4.0a	11.7 ± 1.4a
	White	2.2 ± 2.2b	6.8 ± 0.3b
Z17	Brown	11.1 ± 4.4a	12.5 ± 0.4a
	White	1.1 ± 1.1b	5.4 ± 0.2b

Data are mean ± SE (*n* = 3).

Within a column, data sharing the same lowercase letters are not significantly different at the 0.05 probability level.

The differences in hardness and chewiness were significant between cooked brown and white rice noodles for both X24 and Z17 (Table 4). Cooked brown rice noodles had 25% and 21% lower hardness than cooked white rice noodles for X24 and Z17, respectively. Chewiness was lower in cooked brown rice noodles than in cooked white rice noodles by 27% for X24 and by 26% for Z17. Differences in springiness and cohesiveness were not significant between cooked brown and white rice noodles for either X24 or Z17. There was also no significant difference in resilience between cooked brown and white rice noodles for X24, whereas for Z17 cooked brown rice noodles had 6% lower resilience than cooked white rice noodles.

**Table 4:** Texture profiles of brown and white rice noodles processed from two rice cultivars, Xiangzaoxian 24 (X24) and Zhongjiazao 17 (Z17).

Cultivar	Noodle	Hardness (g)	Chewiness (g)	Springiness (%)	Cohesiveness (%)	Resilience (%)
X24	Brown	2056 ± 83bc	1381 ± 47c	87.7 ± 0.6b	76.7 ± 0.4b	50.1 ± 0.5b
	White	2744 ± 279a	1901 ± 126a	89.2 ± 1.4ab	78.2 ± 1.6ab	54.0 ± 1.1ab
Z17	Brown	1740 ± 103c	1200 ± 78d	88.6 ± 0.5ab	77.8 ± 0.2ab	51.7 ± 0.4b
	White	2197 ± 217b	1614 ± 114b	90.9 ± 0.6a	81.3 ± 1.9a	57.9 ± 1.6a

Data are mean ± SE (*n* = 3).

Within a column, data sharing the same lowercase letters are not significantly different at the 0.05 probability level.

## Discussion

There are few studies comparing the yield of rice noodles processed from brown and white rice, likely because the result is easy to predict. As expected, the higher milling recovery rate of brown rice compared to white rice led to a higher yield of brown rice noodles than white rice noodles in the present study. Furthermore, this study showed that the magnitude of the difference in the milling recovery rate between brown and white rice was comparable to that of the difference in the yield between brown and white rice noodles. This result indicates that the advantage in milling recovery rate of brown rice compared to white rice can be maintained in producing rice noodles. In addition, the results of this study showed that the magnitude of the differences in the milling recovery rate and yield between brown and white rice noodles varied with cultivars: both values were lower in X24 than in Z17. This outcome highlights that the milling property should be listed as a criterion for selecting rice cultivars to produce rice noodles.

Brown rice noodles had poorer cooking quality (higher cooked break rate and cooking loss rate) than white rice noodles. However, both the cooked break rate (about 11%) and the cooking loss rate (about 12%) of brown rice noodles in this study are not high and are within an acceptable range of less than 15%^16^. The poorer cooking quality of brown rice noodles than white rice noodles was attributable to reduced paste viscosities in brown rice flour compared to white rice flour. This is somewhat supported by Bhattachrya et al.^17^, who observed that the cooking loss rate of rice noodles was negatively related to paste viscosities of rice flours. It is well-known that paste properties of rice flour are closely associated with its composition. In particular, Saleh et al.^18^ observed that addition of rice bran into rice flours significantly reduced paste viscosities. Geng et al.^10^ found that peak, trough, and final viscosities were positively related to total starch content in rice flours. Therefore, in this study, the reductions in paste viscosities in brown rice flour compared to white rice flour were attributable to retained rice bran and reduced total starch content.

Cooked brown rice noodles had a softer texture (lower hardness and chewiness) than cooked white rice noodles. This texture property of cooked brown rice noodles should be acceptable to Chinese consumers, who prefer soft-textured rice^6^. It has been long recognized that amylose content is an important factor in determining the texture of cooked rice and rice products^19,20^. Although high amylose content is essential for manufacturing rice noodles, within the range of amylose content that is suitable for manufacturing rice noodles, a lower amylose content can lead to a softer texture of rice noodles. Hence, in this study, the softer texture of cooked brown rice noodles compared to cooked white rice noodles could be partially explained by the lower amylose content in brown rice flour than in white rice flour. More recently, Geng et al.^10^ observed that the chewiness of cooked rice noodles was positively related to total starch content but negatively related to damaged starch, protein, lipid, and dietary fiber contents in rice flours. Hence, in the present study, the lower total starch content in brown rice flour than in white rice flour also resulted in lower chewiness for cooked brown rice noodles compared to cooked white rice noodles.

Although this study did not determine damaged starch, protein, lipid, and dietary fiber contents in rice flours, these ingredients are generally higher in brown rice than in white rice due to lower milling degree^21^. Therefore, the lower milling degree in this study is likely also responsible for the lower chewiness of cooked brown rice noodles compared to cooked white rice noodles. This can be confirmed in future studies by directly comparing damaged starch, protein, lipid, and dietary fiber contents between brown and white rice noodles. In addition, this study showed that there was no significant or consistent difference in elasticity parameters (springiness, cohesiveness, and resilience) between cooked brown and white rice noodles. The inconsistent difference in resilience between cooked brown and white rice noodles across cultivars highlights that the texture quality of brown rice noodles could be improved through cultivar selection.

Taken together, this study suggests that it is feasible to process early-season rice to produce brown rice noodles in terms of yield and quality, and more cultivars should be included in future investigations to obtain more useful information to improve yield and quality of brown rice noodles.

## Methods

Rice grains of two rice cultivars, Xiangzaoxian 24 (X24) and Zhongjiazao 17 (Z17), were collected from the research farm of the Crop and Environment Research Center (28° 09′ N, 113° 37′ E, 43 m asl), at Hunan Agricultural University, China in the early-rice growing season in 2020. The two cultivars were selected because they have high amylose content in rice grains and are widely used to manufacture rice noodles in southern China. The use of plants in the present study complies with international, national and/or institutional guidelines.

Three samples (replicates) of 100 g of rice grains were de-hulled to brown rice and then half of the brown rice was polished to white rice for each cultivar. Samples were weighed to calculate the milling recovery rates of brown and white rice by separately dividing the brown and white rice weight by the grain weight, followed by adjusting moisture content to 0.135 g H_2_O g^−1^.

About 5 g of brown and white rice flours (filtered through 100 mesh) were prepared for each sample to determine starch content and paste properties. Total starch content was determined using an auto digital polarimeter (P850 Pro, Jinan Hanon Instruments Co., Ltd., Jinan, China). Amylose content was measured with the iodine colorimetric method according to the procedure described by Huang et al.^22^. Total starch and amylose contents were adjusted to a moisture content of 0.135 g H_2_O g^−1^. The paste properties (peak, trough, breakdown, final, setback, and consistency viscosities, time to peak viscosity, and paste temperature) were determined using a Rapid Visco Analyzer (RVA-Super 4, Newport Scientific Pty Ltd., Warriewood, Australia).

**Figure 2:**
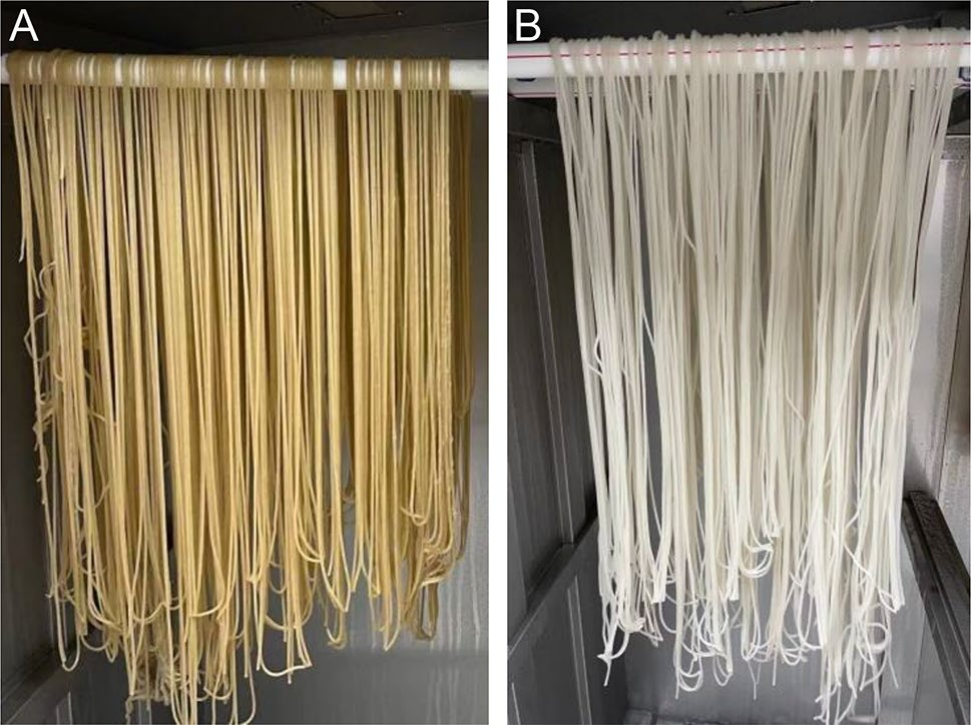
Images showing the brown (**A**) and white rice noodles (**B**) processed in this study.

Six samples of grains were processed to three brown and three white rice samples (500 g per sample) to produce rice noodles. In brief, rice samples were soaked for 10 h at room temperature and then put in an automatic rice noodle-manufacturing machine (5-MFD15B, Hunan Fenshifu Machinery Technology Co., Ltd., Loudi, China) to produce rice noodles with a diameter of 3 mm (Fig. 2A,B). Rice noodles were weighed after they were placed in a self-contained optimization box of the rice noodle-manufacturing machine for 8 h to allow the water to be evenly distributed in the noodles. The yield of rice noodles was calculated by dividing the rice noodle weight by the grain weight.

Thirty noodles with a length of 20 cm were weighed and then boiled in distilled water for 7 min to determine cooking properties (cooked break rate and cooking loss rate) and texture profiles (hardness, springiness, cohesiveness, resilience, and chewiness). Broken noodles were counted to calculate the cooked break rate (number of broken noodles/total number of noodles × 100). Solids lost to the cooking water were oven-dried at 105 °C to a constant weight to calculate the cooking loss rate according to the formula described by Tong et al.^23^. The texture profiles of cooked rice noodles were determined using a texture analyzer (Rapid TA^+^, Shanghai Tengba Instrument Technology Co. Ltd., Shanghai, China).

Data were subjected to analysis of variance followed by the LSD test at the 0.05 probability level (Statistix 8.0, Analytical Software, Tallahassee, Florida, USA).

## Data Availability

All data generated or analysed during this study are included in the article.
